# Repetitive DNAs and differentiation of the ZZ/ZW sex chromosome system in the combtail fish *Belontia hasselti* (Perciformes: Osphronemidae)

**DOI:** 10.1186/s12862-025-02358-y

**Published:** 2025-03-18

**Authors:** Alan Moura de Oliveira, Geize Aparecida Deon, Alexandr Sember, Caio Augusto Gomes Goes, Weerayuth Supiwong, Alongklod Tanomtong, Fábio Porto-Foresti, Ricardo Utsunomia, Thomas Liehr, Marcelo de Bello Cioffi

**Affiliations:** 1 Departamento de Genética e Evolução, Universidade Federal de São Carlos, São Carlos São Paulo, 13565-905 Brazil; 2 Laboratory of Fish Genetics, Institute of Animal Physiology and Genetics, Czech Academy of Sciences, Rumburská, 89, Liběchov, 277 21 Czech Republic; 3 Departamento de Ciências Biológicas, Faculdade de Ciências, Universidade Estadual Paulista, Bauru São Paulo, 17033-360 Brazil; 4 Faculty of Interdisciplinary Studies, Khon Kaen University Nong Khai Campus, Muang, Nong Khai 43000 Thailand; 5 Department of Biology, Faculty of Science, Khon Kaen University, Muang, Khon Kaen 40002 Thailand; 6 Jena University Hospital, Friedrich Schiller University, Institute of Human Genetics, 07747 Jena, Germany

**Keywords:** Sex chromosome evolution, Molecular cytogenetics, Teleostei, Fishes, Satellitome, Isochromosome

## Abstract

**Background:**

Java combtail fish *Belontia hasselti* (Cuvier, 1831), a member of the Osphronemidae family, inhabits lakes and rivers throughout Southeast Asia and Sri Lanka. Previous cytogenetic research revealed it possesses a diploid chromosome number of 48 chromosomes with a female-heterogametic ZZ/ZW sex chromosome system, where the W chromosome is distinguishable as the only metacentric element in the complement. Female-heterogametic sex chromosome systems seem to be otherwise surprisingly rare in the highly diverse order Perciformes and, therefore, *B. hasselti* provides an important comparative model to evolutionary studies in this teleost lineage. To examine the level of sex chromosome differentiation in *B. hasselti* and the contribution of repetitive DNAs to this process we combined bioinformatic analyses with chromosomal mapping of selected repetitive DNA classes, and comparative genomic hybridization.

**Results:**

By providing the first satellitome study in Perciformes, we herein identified 13 satellite DNA monomers in *B. hasselti*, suggesting a very low diversity of satDNA in this fish species. Using fluorescence in situ hybridization, we revealed detectable clusters on chromosomes only for four satellite DNA monomers. Together with the two mapped microsatellite motifs, the repeats primarily accumulated on autosomes, with no distinct clusters located on the sex chromosomes. Comparative genomic hybridization showed no region with accumulated female-specific or enriched repeats on the W chromosome. Telomeric repeats terminated all chromosomes, and no additional interstitial sites were detected.

**Conclusion:**

These data collectively indicate a low degree of sex chromosome differentiation in *B. hasselti* despite their considerable heteromorphy. Possible mechanisms that may underlie this pattern are discussed.

## Introduction

Sex chromosomes, the most prevalent form of genetic sex determination in eukaryotes, evolve through progressive loss of recombination and sequence differentiation. This process displays variable pace among diverse clades. It might result in morphologically different (i.e. heteromorphic) sex chromosome counterparts while in some other cases the sex chromosomes remain (not necessarily due to short evolutionary time) undifferentiated and cytologically indistinguishable (i.e. homomorphic) [1]. Despite over a century of intense research into sex chromosome evolution in numerous eukaryotic lineages, many long-standing questions remain unresolved [1–6]. Teleost fishes provide a broad spectrum of important models to tackle them thanks to the immense variability of sex chromosome systems, their degree of differentiation, and the variability of these patterns at various taxonomic levels, including between populations of the same species in many cases [7–10]. Regarding the female-heterogametic ♂ZZ/♀ZW sex chromosome systems, besides the minority of the homomorphic ones described (e.g. in *Seriola* fishes [11–13] or several cichlids [14, 15]), a considerable portion of reported cases is highly heteromorphic, with the most studied examples concentrated in Neotropical ichthyofauna (e.g., genera *Characidium*, *Parodon*, *Apareiodon*, *Megaleporinus*, and *Triportheus*; e.g [16–21]). Given the long tracts of highly repetitive regions, only recently a more detailed genomic insight has been performed in Neotropical freshwater fishes with highly heteromorphic ZW sex chromosomes [22, 23]. To fully understand the range of variability regarding the sex chromosome evolution and sex-determining pathways in teleosts, more ZW systems should be investigated in various lineages. A particularly intriguing question is: what factors may contribute to their greater differentiation via repetitive DNA accumulation in comparison to established XY and XY-derived systems? In this context, the order Perciformes is particularly interesting, with a highly biased incidence of male-heterogametic systems [7]. In addition, some of the few ZW systems proposed to exist within this species-rich teleost order have been questioned [24] or speculative [25]. Thus, strictly speaking, besides the report of *Epinephelus tauvina* (Fabricius, 1775) [26], the only perciform fish to exhibit heteromorphic ZW sex chromosomes is the Java combtail fish *Belontia hasselti* (Cuvier, 1831) [27].

Belontiinae represents one of the four subfamilies within Osphoronemidae – a lineage that comprises altogether 136 species placed in 14 genera, distributed from Pakistan and India to Southeast Asia [28]. The Belontiinae subfamily includes a single genus *Belontia*, which involves two species: *B. signata* (Günther, 1861) found in Sri Lanka and *B. hasselti* found in southern Thailand, the Malay Peninsula, Sumatra, Java, and Borneo [29, 30]. *Belontia hasselti* is specifically adapted to survive in acidic blackwaters and it also represents a commercially valuable fish commonly found in the aquarium trade. The sole cytogenetic investigation undertaken in this species revealed a diploid chromosome number (2n) of 48 almost exclusively acrocentric chromosomes and the presence of a highly heteromorphic ZW sex chromosome system, where the female-limited W chromosome is the only metacentric element in the karyotype [27].

Various repetitive DNA classes have been found accumulated on highly heteromorphic sex chromosomes in teleosts, such as microsatellites (e.g [19, 31–35]), ribosomal RNA genes (e.g [20, 36–38]), transposable elements (e.g [18, 35, 39–41]), telomeric repeats [35], and especially in the recent years also satellite DNAs (satDNA) [42–48]. Repetitive DNAs are therefore intimately associated with sex chromosome evolution and may also directly contribute to master sex-determining gene establishment [49–51] or its relocation to other chromosomal pair in the process referred to as sex chromosome turnover (e.g., in salmonid fishes – reviewed in [52]).

Satellite DNA represents one of the most abundant and variable repetitive DNA fractions in the eukaryotic genomes, both in terms of sequence diversity and patterns of organization across chromosomes. The entire collection of satDNA monomers per species is being termed satellitome and, given its fast evolution, the satellitome properties may vary greatly even between closely related species [53–55]. Among teleosts, the link between satDNAs and sex chromosome evolution has thus far been traced mostly among Neotropical members of the orders Characiformes and Siluriformes ([42, 43, 45, 47, 48, 56, 57] among others) but analogous studies have also been undertaken in African annual killifishes [44, 46]. The satellitomes in the context of ZZ/ZW sex chromosome evolution in Neotropical fishes have been studied thoroughly in the genus *Triportheus* [43, 57] and *Megaleporinus* [42, 56]. In both cases, the W chromosomes are heterochromatin-rich and display a high concentration of satDNAs [42, 43, 56, 57].

In this study, we integrated cytogenetic and genomic methods to examine the patterns of evolution and differentiation in the ZW sex chromosome system of *B. hasselti*. To this end, we characterized the *B. hasselti* satellitome (being thus the first analyzed perciform species in this respect) and performed chromosomal mapping of the satDNA monomers alongside some other tandemly repeated sequences, namely telomeric repeats and microsatellites. Lastly, we also assessed the degree of sex chromosome differentiation using comparative genomic hybridization (CGH). Our results revealed the lowest satDNA monomer diversity among the teleosts analyzed to date and a low level of ZW sex chromosome differentiation.

## Results

### Satellitome characterization

**Fig. 1:**
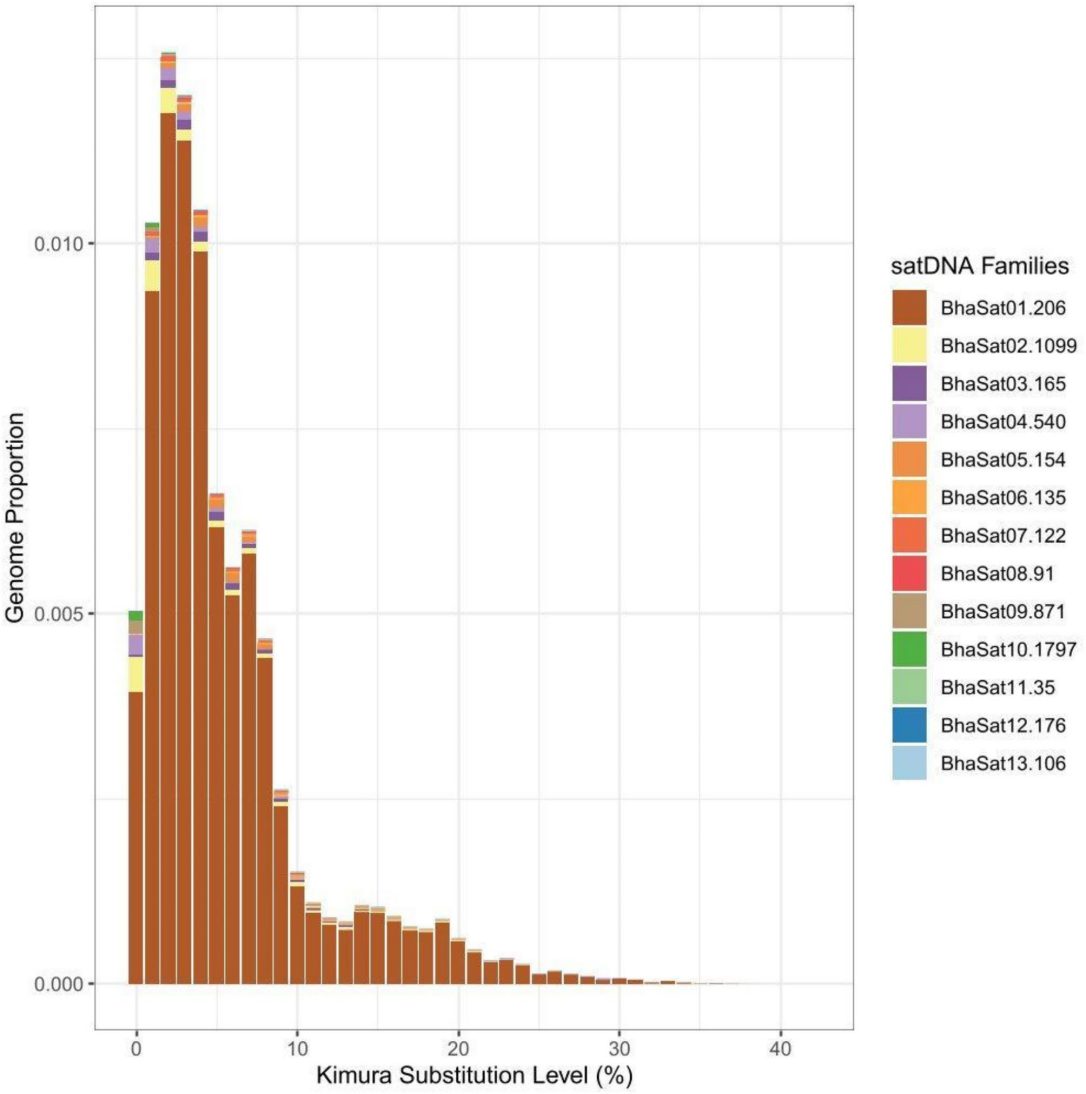
Repeat landscapes showing the abundance and divergence profiles for all satDNA monomers identified in the *Belontia hasselti* genome

The iterations carried out using the Tandem Repeat Analyzer (TAREAN) software resulted in a total of 13 satDNA families for *B. hasselti*, hereafter named as BhaSatDNAs (Table 1). The vast majority of the satellites found are characterized as long (> 100 bp), with the lengths of their repeat units (monomers) varying between 35 bp and 1797 bp. The A + T content ranged from 38.70 to 68.70% with an average of 51.4%. Divergence ranged from 0.73 to 15.11%, with an average of 6.83% among the satDNAs. The repeat landscapes generated from the analyses are shown in Fig. 1.

**Table 1:** General features of *Belontia. hasselti* satellitome, including the monomer sizes in base pairs (bp), abundance, divergence, and A + T content

satDNA family	Monomer size (bp)	Abundance	Divergence (%)	A + T (%)
BhaSat01-206	206 bp	0,081792517	5,73	60,70%
BhaSat02-1099	1099 bp	0,002210017	4,5	57,30%
BhaSat03-165	165 bp	0,001078853	6,12	55,80%
BhaSat04-540	540 bp	0,000974244	4,11	59,80%
BhaSat05-154	154 bp	0,000770163	5,37	36,40%
BhaSat06-135	135 bp	0,000470657	10,36	57,80%
BhaSat07-122	122 bp	0,000385905	6,43	55,70%
BhaSat08-91	91 bp	0,000274007	9,44	62,60%
BhaSat09-871	871 bp	0,000267607	0,73	45,80%
BhaSat10-1797	1797 bp	0,000249389	1,64	53,40%
BhaSat11-35	35 bp	0,000215946	15,11	54,30%
BhaSat12-176	176 bp	9.01e-05	11,96	68,70%
BhaSat13-106	106 bp	5.51e-05	7,35	38,70%

### Fluorescence in situ hybridization (FISH)

**Fig. 2:**
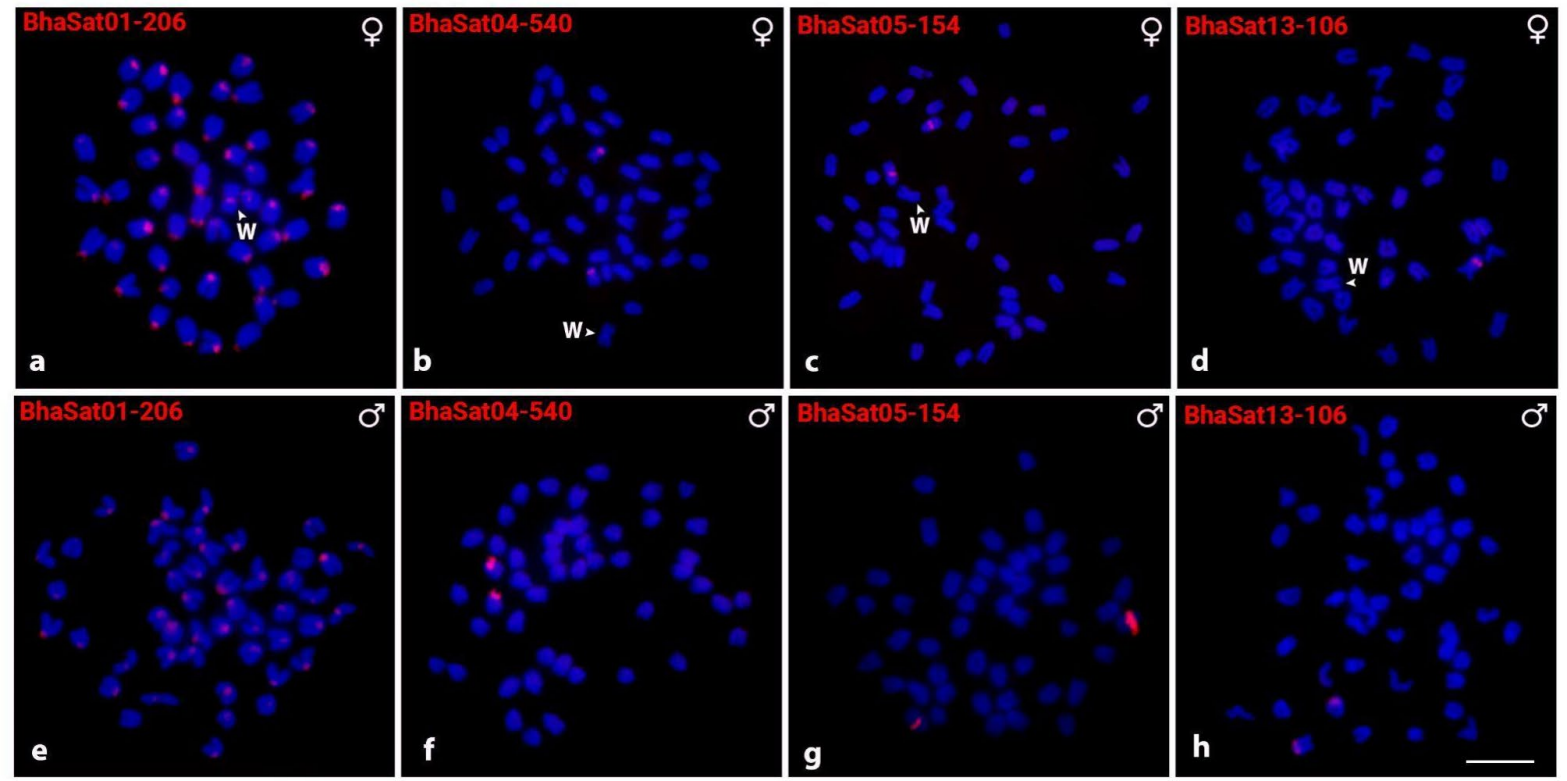
Metaphase chromosome plates of *Belontia hasselti* after in situ hybridization experiments with BhaSatDNAs in female (**a**-**d**) and male (**e**-**h**) individuals. The satDNA family names are indicated in the upper left corner. The arrowhead indicates the W chromosome. Scale bar = 10 μm

Only four out of 13 characterized BhaSatDNA monomers displayed positive FISH signals on *B. hasselti* chromosomes (Fig. 2). The BhaSat01-206 was shown to accumulate in the (peri)centromeric regions of all chromosomes, while the BhaSat04-540 and BhaSat05-154 monomers each located to a single chromosome pair (18 and 5 respectively), with no difference observed between male and female individuals. In contrast, the BhaSat13-106 exhibited sex-linked variation in the distribution pattern, where males possessed signals on both homologs of a single chromosome pair, while females displayed the signal on just one homolog. Though identifying the Z chromosome is challenging, it is nonetheless plausible to infer that this satDNA is associated with the Z chromosome, given this pattern was consistent across all individuals investigated (Fig. 2).

### C-banding and FISH with telomeric and microsatellite probes

**Fig. 3:**
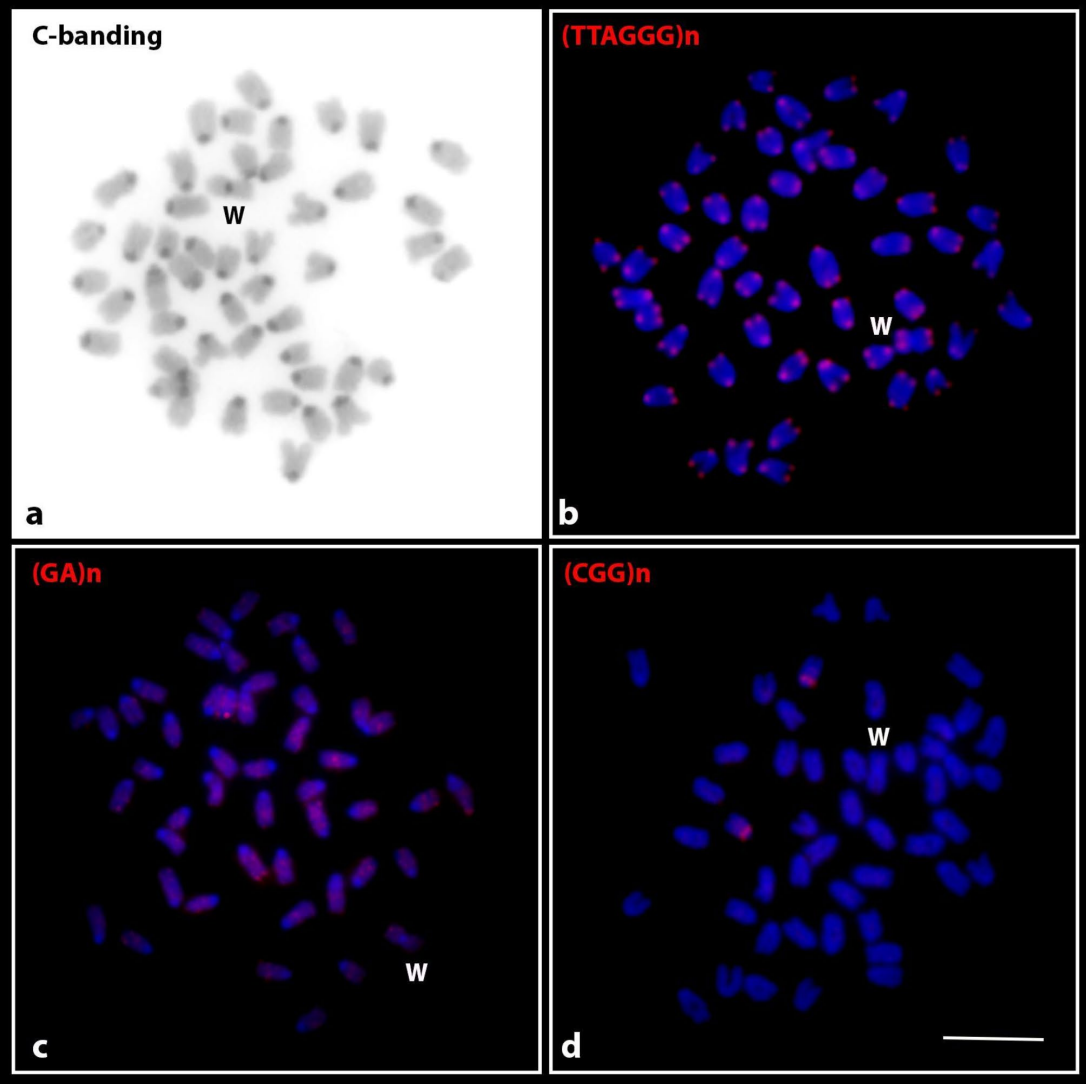
Female metaphase chromosomes of *Belontia hasselti* after C-banding (**a**) and fluorescence in situ hybridization with telomeric (**b**) and microsatellite sequence motifs (GA)_n_ (**c**) and (CGG)_n_ (**d**). The probe used is indicated in the top left corner. The W sex chromosome is marked. Scale bar = 10 μm

Microsatellite mapping on the chromosomes of *B. hasselti* resulted in positive signals for the (GA)_n_ and (CGG)_n_ sequences. Detectable (GA)_n_ arrays were scattered across several chromosomes except for the (peri)centromeric regions (Fig. 3c), while the (CGG)_n_ motif was localized in the (peri)centromeric region of only a single chromosome pair (Fig. 3d). Constitutive heterochromatin was detected in all chromosomes, mostly in pericentromeric regions (Fig. 3a). Telomeric sequences were identified at the terminal regions of all chromosomes. No additional (interstitial) sites were observed.

### Patterns of comparative genomic hybridization (CGH)

**Fig. 4:**
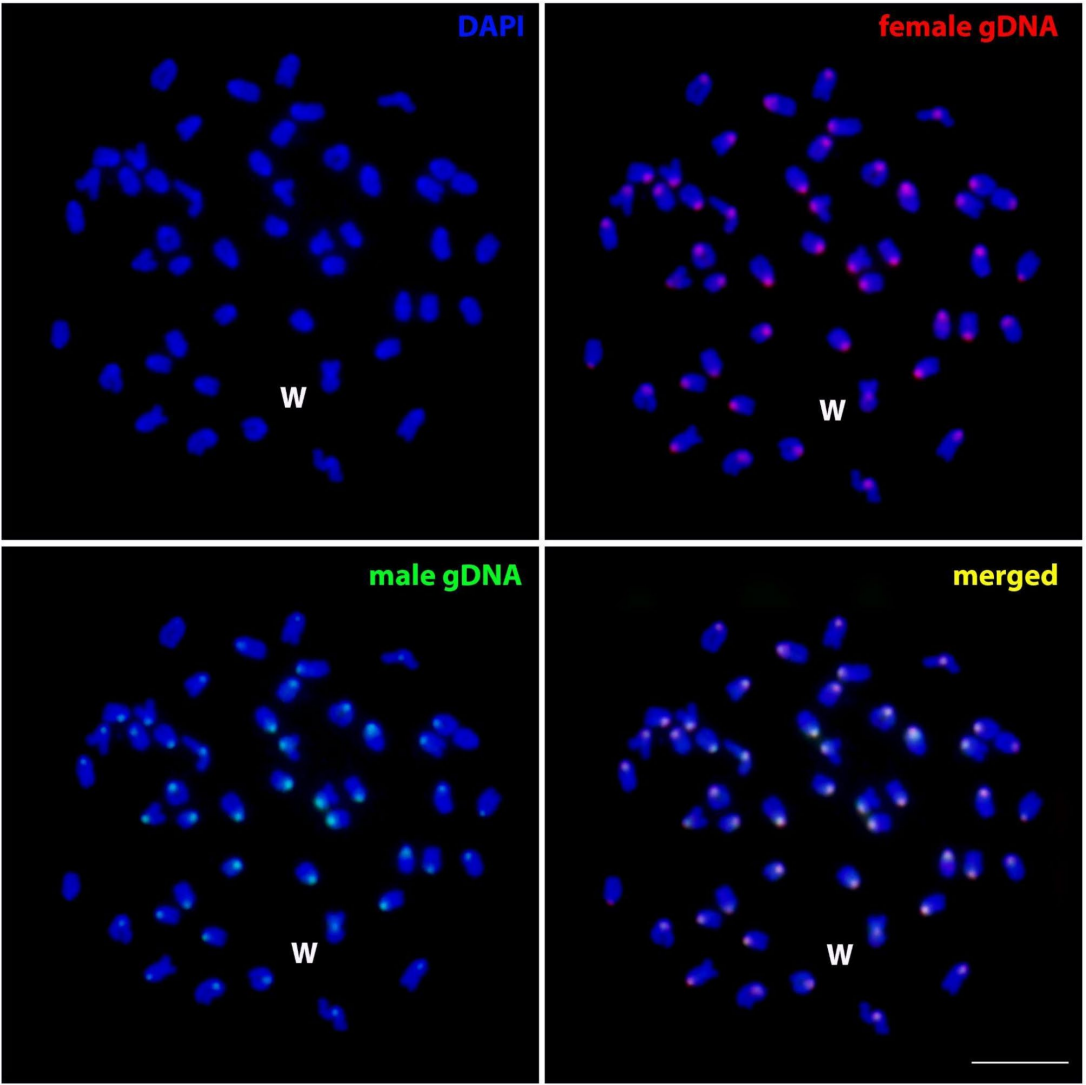
Female metaphase spread of *Belontia hasselti* after comparative genomic hybridization. Female (red; Atto-550-dUTP-labeled) and male (green; Atto-488-dUTP-labeled) genomic DNA along with DAPI chromosome counterstaining (blue).The merged image with DAPI staining and both genomic probes (indicated in yellow) shows no biased or specific signals of either of the probes. The W chromosome is indicated. Scale bar = 10 μm

Comparative genomic hybridization analysis of male and female individuals of *B. hasselti* revealed overlapping signals especially in the (peri)centromeric regions of all chromosomes. No regions marked exclusively by either of the genomic probes were observed on metaphases within our sampling (Fig. 4).

## Discussion

By analysing the combtail fish *B. hasselti*, the present study provides the first satellitome analysis in the highly diverse teleost order Perciformes, with a special emphasis on the link between satDNA and sex chromosome evolution in this species. The genome proportion of satDNAs varies considerably among related species in diverse animal and plant lineages [55]. In teleost fishes, the largest satDNA libraries identified to date harbor 140 and 164 different satDNA monomers, presented in the two Anostomidae species, *Megaleporinus elongatus* (Valenciennes, 1850) and *M. macrocephalus* (Garavello & Britski, 1988), respectively [42, 58]. On the opposite side of the spectrum, the smallest satellitome to date (25 monomers) has been reported in the Asian arowana *Scleropages formosus* (Müller & Schlegel, 1840) [59]. In the present study, we provide the new lowest teleost satellitome, composed of only 13 satDNA monomers (BhaSatDNAs) in *B*. *hasselti*. The satDNA fraction builds up only 8.86% of the genome of this fish.

Eight out of thirteen BhaSatDNAs did not display visible signals after FISH, most probably due to their non-clustered organization and/or low abundance in the genome, hampering thereby the generation of signals above the method’s detection limit [60–62]. The remaining four BhaSatDNAs (BhaSat01-206, BhaSat04-540, BhaSat05-154, and BhaSat13-106) displayed a range of distinct detectable patterns on chromosomes after FISH (Fig. 2). The most abundant monomer, BhaSat01-206, was detected in the (peri)centromeric regions of all chromosomes (Fig. 2). This pattern together with this monomer being the most abundant satDNA in the *B. hasselti* genome suggest that this satDNA might play a role in centromere organization and/or function. In teleosts, the prime candidates for centromeric satDNA with these features have been so far identified e.g. in two Neotropical characiform species, *Triportheus auritus* (Valenciennes, 1850) (TauSat01-119; [43]), and *Prochilodus lineatus* (Valenciennes, 1837) (PliSat01-225; [63]), and further in African *Nothobranchius* killifishes [44, 64]. However, further evidence from other Neotropical species strongly suggests that less abundant satDNAs may also take over the centromere-related role [48, 65, 66]. In the case of BhaSat01-206 of *B. hasselti*, direct experimental testing of the interaction between this satDNA and core centromeric protein CENP-A needs to be undertaken in future studies.

The other two mapped satDNAs, BhaSat04-540 and BhaSat05-154, formed (peri)centromeric and interstitial clusters, respectively, on a single autosome pair (Fig. 2). Interestingly, the last monomer with identifiable signals, BhaSat13-106, presented sex-linked differences in the hybridization pattern, with a signal on both homologs of a single chromosome pair in males but on a single homolog in females. Due to its highly dynamic nature, satDNA evolution is frequently associated with the amplification and contraction of tandem arrays, along with the possible dissemination of the repeats to other genomic locations [54, 55, 60, 67, 68]. It is therefore reasonable to assume that the obtained pattern might reflect the presence/absence polymorphism due to the variable degree of repeat cluster size among homologs, and eventually entire cluster elimination from one of the homologs. The sex-related pattern is puzzling; although our findings align with the hypothesis that this satDNA may represent a marker exclusive to the Z chromosome, the difficulty in identifying the Z chromosome (since it has similar size to the autosomes) using chromosomal markers does not allow us to fully confirm this interpretation.

Teleost species exhibiting a predominantly heteromorphic ZW sex chromosome system typically possess a significantly larger W chromosome compared to its Z counterpart, resulting from considerable accumulation and/or amplification of repetitive DNA, characterized by an extensive block of heterochromatin. A shrunk, largely degenerated W is also present in several lineages, however (reviewed in [7, 69]). Satellitome investigations conducted on the ZW system in fishes indicate fundamental role of this repetitive DNA class in sex chromosome differentiation. In the highly differentiated ZW system of *M. macrocephalus*, for instance, out of 22 satDNAs evidenced to accumulate on the W chromosome, 14 monomers were exclusively located only on this female-limited sex chromosome [42]. Previous formal analyses based on the updated list of fish sex chromosome systems provided significant support for the hypothesis that, in teleost fishes, female-heterogametic systems generally differentiate faster than male-heterogametic ones [7]. The observed patterns in *B. hasselti*, however, reveal an unusual case of significantly heteromorphic, enlarged W chromosome, yet characterized by a low degree of heterochromatinization, confined to the pericentromeric region ([27]; present study). Such a discrepancy between apparent heteromorphism (considerably enlarged W chromosome) and absence of large blocks of heterochromatin has been previously described among teleosts in Western mosquitofish *Gambusia affinis* (Baird & Girard 1853) [69]. More recent study in this fish indicated that despite this fact, the W chromosome is densely populated with microsatellites and transposable elements [70]. Further study is therefore required to expand the analysis of W-linked patterns in *B. hasselti* by other repetitive DNA classes. Thus far, our CGH assay additionally suggested that qualitative differences in repeat content between Z and W might not be pronounced in *B. hasselti*, as both male and female genomic probe co-hybridized equally across the chromosome complement, with predominant accumulations in the (peri)centromeric regions only (Fig. 4). These findings imply that either the region of differentiation between Z and W is physically small and cannot be detected at the resolution of CGH, or, alternatively, the lack of sex-specific signal might reflect insufficient differences in the amount and composition of accumulated repetitive DNA across the chromosome complement between sexes (cf. [44, 71]). In summary, together with the low level of constitutive heterochromatin and accumulated repeats evidenced herein and also by Chaiyasan et al. [27], the results indicate a low degree of Z-W sequence divergence in *B. hasselti*.

Another possible mechanism explaining the occurrence of the enlarged, yet non-heterochromatinized W chromosome might be amplification of gene copies, which creates tandemly repeated segments (amplicons). An increasing number of studies in diverse eukaryotic lineages suggest that this mechanism might represent a common feature accompanying sex chromosome differentiation (e.g [72–75]). A deeper gene content analysis of the W chromosome will determine whether this mechanism might hold also for *B. hasselti*.

We cannot further exclude the possibility that the addition of another chromosome material (via fusion or translocation event) might enlarge the *B. hasselti* W chromosome similarly as it was described in certain cichlid fish species of the Oreochromini tribe [76]. The absence of interstitial telomeric signals (ITS) does not corroborate this reasoning, however, it is also probable that telomeric sequences might have been lost prior to chromosome fusion or, alternatively, DNA repair mechanisms might have removed telomeric repeats from the fusion breakpoint.

The evident symmetry of both W chromosome arms regarding their length and the absence of ITS led us to assume yet another and perhaps slightly more plausible scenario for the origin of the W chromosome – through the isochromosome formation. This might have proceeded via aberrant centromere division during mitosis or meiosis, resulting in the separation of chromosome arms rather than chromatids. The symmetric location of repetitive DNA classes, mostly satDNAs, on both chromosome arms of a metacentric chromosome has served as a supporting evidence for this mechanism, proposed previously to originate sex chromosomes in *Rumex acetosa* [77] and B chromosomes in several Neotropical fish species [65, 78–81]. In favor of this possibility, none of the cytogenetic patterns previously reported for *B. hasselti* [27] and in the present study show clearly asymmetric distribution between the W chromosome arms. What is more, a slightly supportive pattern has been obtained by mapping the (CA)_15_ microsatellite motif which tentatively suggests symmetrical distribution across the W chromosome arms [27]. However, given the widespread distribution of this microsatellite throughout the entire chromosome complement, this pattern may equally likely reflect the exclusion of this microsatellite motif from centromeres. Studies involving whole chromosome painting probes and closely related species might provide important data for testing the above hypotheses.

## Conclusion

The satellitome of *B. hasselti*, first one studied within the order Perciformes, turned out to encompass the lowest number of satDNA monomers described in teleosts to date. A subset of satDNA monomers provided detectable signals on the chromosomes and their patterns, together with those from mapping other repetitive sequences and the CGH method, reinforce the view that the ZW sex chromosome system in *B. hasselti* shows little differentiation at the level of repetitive DNA accumulation (although limitations of our approach have been discussed), despite its highly heteromorphic character with large euchromatic W chromosome. *Belontia hasselti* thus provides a promising model for investigating alternative hypotheses which could explain the unusual pattern of sex chromosome differentiation in the perciform genome with low satDNA diversity.

## Materials and methods

### Chromosomal Preparation and C-banding

Ten individuals (five of each sex) of *B. hasselti* were collected from the To Daeng peat swamp forest in Narathiwat Province, Thailand. Metaphase chromosomes were obtained from kidney using the colchicine treatment as described in [82] and the general protocol following Supiwong et al. [83]. This species is classified as Least Concern (LC) on the IUCN Red List, thus no permissions were necessary for specimen collection under the local legislation. All the procedures followed ethical protocols approved by the Institutional Animal Care and Use Committee of Khon Kaen University, based on the Ethics of Animal Experimentation of the National Research Council of Thailand (ACUC-KKU-90/60). Constitutive heterochromatin distribution was visualized by C-banding according to standard protocol [84].

### DNA extraction and genome sequencing

The phenol-chloroform-isoamyl alcohol method [85] was applied to extract genomic DNAs (gDNAs) from female and male individuals. The samples were subsequently sequenced on the BGISEQ-500 platform at BGI (BGI Shenzhen Corporation, Shenzhen, China).

### Characterization of *Belontia hasselti* satellitome

The characterization of *B. hasselti* satellitome was carried out using the TAREAN software [86], available on the Galaxy platform (https://repeatexplorer-elixir.cerit-sc.cz/galaxy). Firstly, raw reads were quality-filtered using the Trimmomatic software [87] with Q < 20, using the options LEADING:3 TRAILING:3 SLIDINGWINDOW:4:20 MINLEN: 100 CROP: 101 bp parameters. Then, 2 × 500,000 reads were randomly selected for clustering in TAREAN. After this, the putative satellite DNAs identified by TAREAN were filtered from the original library using the DeconSeq software [88] and a new sampling of 2 × 500,000 reads was carried out, with subsequent clustering by TAREAN.

The filtering and re-clustering process was repeated until no putative satellite DNA was recovered by TAREAN. After this, we filtered out multigene families from the catalog of satDNAs. For this, any sequence that exhibited at least 50% similarity to any multigene DNA family (e.g., ribosomal DNA classes) was discarded. In addition, a homology search was carried out using the RepeatMasker software, implemented in a custom Python script (https://github.com/fjruizruano/satminer/blob/master/rm_homology.py) to check for possible redundancies, comparing the similarity between each satDNA sequence. This process used the previously proposed parameters [60], where sequences with over 95% similarity were considered variants of the same satellite, those with between 80% and 95% similarity were considered part of the same satellite DNA family, and those with between 50% and 80% similarity were considered superfamilies. The catalogs were deposited on GenBank with the accession numbers PQ062508–PQ062520.

### Abundance and diversity of SatDNAs

The abundance and divergence of *B. hasselti* satDNA monomers were calculated using the RepeatMasker software [89] using the “cross_match” tool. For this, a sample of 2 × 5,000,000 reads from each library were used. The abundance of each satDNA was obtained by dividing the number of mapped reads by the sum of the total nucleotides. In this way, the satDNAs were ranked in decreasing order of abundance, renamed with the abbreviation of the species name (Bha), together with the term “Sat” followed by the catalog number and the size of the repeated unit (monomer size), as suggested by Ruiz-Ruano et al. [60]. Also, through RepeatMasker, using the Kimura-2 parameter based on the calcDivergenceFromAlign.py script [89], the genetic distance between the BhaSatDNAs was calculated and then represented in a Repeat landscape.

### Primer design and DNA amplification by polymerase chain reaction

After isolating the catalog of satellite DNAs, specific primers were manually designed, corresponding to eleven of the thirteen families of satDNAs isolated from *B. hasselti*. BhaSat11-35, due to its small motif size, was synthesized directly conjugated with Biotin-16-dUTP. As for BhaSat08, due to self-ringing problems, primers were not synthesized. For the eleven satDNA sequences, amplifications were carried out using polymerase chain reaction (PCR), with the reaction conditions and thermal profiles optimized according to Štundlová et al. [44]. The following cycle was used: (i) initial denaturation of 95 ºC for 5 min, (ii) 34 cycles with a denaturation step of 95 ºC for 40 s; an annealing step varying from 52 ºC to 61 ºC for 40 s, depending on the satDNA; and an extension step of 72 ºC for 45 s, (iii) final extension of 72 ºC for 10 min. Once this stage had been completed, the PCR products were checked in a 2% agarose gel to ensure the amplification and integrity of the satDNAs. Finally, these products were quantified using a NanoDrop 2000/2000c spectrophotometer (Thermo Fisher Scientific, Waltham, USA).

### Probe labeling and fluorescence in situ hybridization

Once the amplifications had been carried out, the PCR products were labeled using the nick-translation technique via the Jena Bioscience Kit (Jena, Germany), in which the Atto-550-dUTP nucleotide was incorporated into the satDNA sequence according to the manufacturer’s protocol. Microsatellite sequences directly labeled with Cy3 at the 5' end during the synthesis (VBC Biotech, Vienna, Austria) [(GA)_n_, (TA)_n_, (GAG)_n_, (CGG)_n_, (CAT)_n_, (TAC)_n_, (C30)_n_, (A30)_n_, (GC)_n_] and telomeric repeats (TTAGGG)_n_ isolated according to [90] and labeled with Atto-550-dUTP through nick translation (Jena Bioscience) were also used as probes. Fluorescence in situ hybridization experiments were carried out on the chromosomes of females and males of the *B. hasselti* species following the protocol described in [91].

### Assessment of genetic composition of sex chromosomes by comparative genomic hybridization

The genomic DNA of male and female *B. hasselti* was labeled with Atto-488-dUTP and Atto-550-dUTP, respectively, using the nick-translation kit from Jena Bioscience. Unlabeled C0t-1 DNA, prepared from male gDNA following the protocol of [92], was added to the probe to reduce the hybridization of shared abundant repeats. Thus, the final probe mix for one slide consisted of 3 µg of male-derived C0t-1 DNA and 500 ng of each labeled male- and female-derived gDNAs. After precipitation with 100% ethanol, the probes were air-dried and the pellets resuspended in a hybridization buffer (20 µl per slide), which contained 50% formamide, 2× SSC, and 10% dextran sulfate. The FISH experiments for CGH followed the methodology described in [93].

### Microscopy and image analysis

A minimum of 30 metaphase spreads were examined per individual to ascertain the FISH results. Images were taken with a CoolSNAP camera attached to an Olympus BX50 microscope (Olympus Corporation, Ishikawa, Japan), and then processed using Image-Pro Plus 4.1 software from Media Cybernetics (Silver Spring, Maryland, USA).

## Data Availability

The datasets generated during and/or analyzed during the current study are available in the GenBank database (https://www.ncbi.nlm.nih.gov/genbank/) under accession numbers PQ062508–PQ062520. All the data generated or analyzed during this study are included in this article.

## Abbreviations

satDNA: Satellite DNA
CGH: Comparative genomic hybridization
TAREAN: Tandem Repeat Analyzer
Bp: Base pair
BhaSatDNAs: *Belontia hasselti* Satellite DNA
FISH: Fluorescence in situ hybridization
DAPI: 4’,6-diamidino-2-phenylindole
C-banding: Constitutive heterochromatin banding
gDNAs: Genomic DNAs
PCR: Polymerase chain reaction
ITS: Interstitial telomeric sites

## References

[1] Charlesworth D. When and how do sex-linked regions become sex chromosomes? Evolution. 2021; 10.1111/evo.14196 10.1111/evo.14196 33592115

[2] Abbott JK, Nordén AK, Hansson B. Sex chromosome evolution: historical insights and future perspectives. Proc R Soc B. 2017. 10.1098/rspb.2016.2806. 10.1098/rspb.2016.2806 PMC5443938 28469017

[3] Vicoso B. Molecular and evolutionary dynamics of animal sex-chromosome turnover. Nat Ecol Evol. 2019. 10.1038/s41559-019-1050-8. 31768022 10.1038/s41559-019-1050-8

[4] Furman BL, Metzger DC, Darolti I, Wright AE, Sandkam BA, Almeida P, et al. Sex chromosome evolution: so many exceptions to the rules. Genome Biol Evol. 2020. 10.1093/gbe/evaa081. 32315410 10.1093/gbe/evaa081 PMC7268786

[5] Jay P, Jeffries D, Hartmann FE, Véber A, Giraud T. Why do sex chromosomes progressively lose recombination? Trends Genet. 2024. 10.1016/j.tig.2024.03.005. 38677904 10.1016/j.tig.2024.03.005

[6] Saunders PA, Muyle A. Sex chromosome evolution: hallmarks and question marks. Mol Biol Evol. 2024. 10.1093/molbev/msae218. 39417444 10.1093/molbev/msae218 PMC11542634

[7] Sember A, Nguyen P, Perez MF, Altmanová M, Ráb P, Cioffi MB. Multiple sex chromosomes in teleost fishes from a cytogenetic perspective: state of the art and future challenges. Phil Trans R Soc B: Biol Sci. 2021. 10.1098/rstb.2020.0098. 10.1098/rstb.2020.0098 PMC8310710 34304595

[8] Lichilín N, Salzburger W, Böhne A. No evidence for sex chromosomes in natural populations of the cichlid fish *Astatotilapia burtoni*. G3 (Bethesda). 2023. 10.1093/g3journal/jkad011. 10.1093/g3journal/jkad011 PMC9997565 36649174

[9] Smith SH, Hsiung K, Böhne A. Evaluating the role of sexual antagonism in the evolution of sex chromosomes: new data from fish. Curr Opin Genet Dev. 2023. 10.1016/j.gde.2023.102078. 37379742 10.1016/j.gde.2023.102078

[10] Kitano J, Ansai S, Takehana Y, Yamamoto Y. Diversity and convergence of sex-determination mechanisms in teleost fish. Annu Rev Anim Biosci. 2024. 10.1146/annurev-animal-021122-113935. 37863090 10.1146/annurev-animal-021122-113935

[11] Purcell CM, Seetharam AS, Snodgrass O, Ortega-García S, Hyde JR, Severin AJ. Insights into teleost sex determination from the *Seriola dorsalis* genome assembly. BMC Genomics. 2018. 10.1186/s12864-017-4403-1. 29310588 10.1186/s12864-017-4403-1 PMC5759298

[12] Koyama T, Nakamoto M, Morishima K, Yamashita R, Yamashita T, Sasaki K, et al. A SNP in a steroidogenic enzyme is associated with phenotypic sex in *Seriola* fishes. Curr Biol. 2019. 10.1016/j.cub.2019.04.069. 31130458 10.1016/j.cub.2019.04.069

[13] Li M, Xu X, Liu S, Fan G, Zhou Q, Chen S. The chromosome-level genome assembly of the Japanese yellowtail jack *Seriola aureovittata* provides insights into genome evolution and efficient oxygen transport. Mol Ecol Resour. 2022. 10.1111/1755-0998.13648 35593537 10.1111/1755-0998.13648

[14] Gammerdinger WJ, Kocher TD. Unusual diversity of sex chromosomes in African cichlid fishes. Genes. 2018. 10.3390/genes9100480. 30287777 10.3390/genes9100480 PMC6210639

[15] El Taher A, Ronco F, Matschiner M, Salzburger W, Böhne A. Dynamics of sex chromosome evolution in a rapid radiation of cichlid fishes. Sci Adv. 2021. 10.1126/sciadv.abe8215. 34516923 10.1126/sciadv.abe8215 PMC8442896

[16] Bellafronte E, Schemberger MO, Artoni RF, Filho OM, Vicari MR. Sex chromosome system ZZ/ZW in *Apareiodon hasemani* Eigenmann, 1916 (Characiformes, Parodontidae) and a derived chromosomal region. Genet Mol Biol. 2012. 10.1590/S1415-47572012005000077. 10.1590/S1415-47572012005000077 PMC3526084 23271937

[17] Schemberger MO, Oliveira JIN, Nogaroto V, Almeida MC, Artoni RF, Cestari MM, et al. Construction and characterization of a repetitive DNA library in Parodontidae (Actinopterygii: Characiformes): a genomic and evolutionary approach to the degeneration of the W sex chromosome. Zebrafish. 2014. 10.1089/zeb.2014.1013. 25122415 10.1089/zeb.2014.1013 PMC4248244

[18] Schemberger MO, Nascimento VD, Coan R, Ramos É, Nogaroto V, Ziemniczak K, et al. DNA transposon invasion and microsatellite accumulation guide W chromosome differentiation in a Neotropical fish genome. Chromosoma. 2019. 10.1007/s00412-019-00721-9. 31456013 10.1007/s00412-019-00721-9

[19] Scacchetti PC, Utsunomia R, Pansonato-Alves JC, Vicari MR, Artoni RF, Oliveira C, et al. Chromosomal mapping of repetitive DNAs in *Characidium* (Teleostei, Characiformes): genomic organization and diversification of ZW sex chromosomes. Cytogenet Genome Res. 2015. 10.1159/000437165. 26277929 10.1159/000437165

[20] Yano CF, Bertollo LAC, Ezaz T, Trifonov V, Sember A, Liehr T, et al. Highly conserved Z and molecularly diverged W chromosomes in the fish genus *Triportheus* (Characiformes, Triportheidae). J Hered. 2017. 10.1038/hdy.2016.83. 10.1038/hdy.2016.83 PMC5315523 28000659

[21] de Barros CL, Piscor D, Parise-Maltempi PP, Feldberg E. Differentiation and evolution of the W chromosome in the fish species of *Megaleporinus* (Characiformes, Anostomidae). Sex Dev. 2018. 10.1159/000489693. 10.1159/000489693 29879699

[22] Souza-Borges CHD, Utsunomia R, Varani AM, Uliano-Silva M, Lira LVG, Butzge AJ, et al. De novo assembly and characterization of a highly degenerated ZW sex chromosome in the fish *Megaleporinus macrocephalus*. GigaScience. 2024. 10.1093/gigascience/giae085. 39589439 10.1093/gigascience/giae085 PMC11590113

[23] Wolf IR, Schemberger MO, Azambuja M, Oliveira FS, Nogaroto V, Valente GT, et al. The long-read assembly of *Apareiodon* sp., a Neotropical fish with a ZZ/ZW sex chromosome system. Genet Mol Biol. 2024. 10.1590/1678-4685-GMB-2024-0098. 10.1590/1678-4685-GMB-2024-0098 PMC11468460 39392722

[24] Jeffries DL, Mee JA, Peichel CL. Identification of a candidate sex determination gene in *Culaea inconstans* suggests convergent recruitment of an *Amh* duplicate in two lineages of stickleback. J Evol Biol. 2022. 10.1111/jeb.14034. 35816592 10.1111/jeb.14034 PMC10083969

[25] Teal CN, Coykendall DK, Campbell MR, Eardley DL, Delomas TA, Shira JT, et al. Sex-specific markers undetected in green sunfish *Lepomis cyanellus* using restriction‐site associated DNA sequencing. J Fish Biol. 2022. 10.1111/jfb.15063. 35439326 10.1111/jfb.15063

[26] Dass CC. Cited in Rishi KK. Current status of fish cytogenetics. In: Das and Jhingram, editors, Fish Genetics in India, Proc. 70th Indian Sci. Cong. vol. 2. Today and Tomorrow’s Printers; 1983. p. 1.

[27] Chaiyasan P, Mingkwan B, Jantarat S, Suwannapoom C, Cioffi MB, Liehr T, et al. Classical and molecular cytogenetics of *Belontia hasselti* (Perciformes: Osphronemidae): insights into the ZZ/ZW sex chromosome system. Biodiversitas. 2021. 10.13057/biodiv/d220205.

[28] Nelson JS, Grande TC, Wilson MVH. Fishes of the world. Hoboken: Wiley; 2016.

[29] Roberts TR. The freshwater fishes of Western Borneo (Kalimantan Barat, Indonesia). Memoirs of the California Academy of Sciences; 1989.

[30] Pethiyagoda R. Conservation of Sri Lankan freshwater fishes. The fauna of Sri Lanka; 2006. pp. 103–12.

[31] Terencio ML, Schneider CH, Gross MC, Vicari MR, Farias IP, Passos KB, et al. Evolutionary dynamics of repetitive DNA in *Semaprochilodus* (Characiformes, Prochilodontidae): a fish model for sex chromosome differentiation. Sex Dev. 2013. 10.1159/000356691. 24296872 10.1159/000356691

[32] Poltronieri J, Marquioni V, Bertollo LAC, Kejnovsky E, Molina WF, Liehr T, et al. Comparative chromosomal mapping of microsatellites in *Leporinus* species (Characiformes, Anostomidae): unequal accumulation on the W chromosomes. Cytogenet Genome Res. 2014. 10.1159/000355908. 24217024 10.1159/000355908

[33] Ziemniczak K, Traldi JB, Nogaroto V, Almeida MC, Artoni RF, Moreira-Filho O, et al. *In situ* localization of (GATA)n and (TTAGGG)n repeated DNAs and W sex chromosome differentiation in Parodontidae (Actinopterygii: Characiformes). Cytogenet Genome Res. 2014. 10.1159/000370297. 25662193 10.1159/000370297

[34] Yano CF, Bertollo LAC, Liehr T, Troy WP, Cioffi MB. W chromosome dynamics in *Triportheus* species (Characiformes, Triportheidae): an ongoing process narrated by repetitive sequences. J Hered. 2016. 10.1093/jhered/esw021. 27036509 10.1093/jhered/esw021 PMC4888443

[35] Dulz TA, Azambuja M, Lorscheider CA, Noleto RB, Moreira-Filho O, Nogaroto V, et al. Repetitive DNAs and chromosome evolution in *Megaleporinus obtusidens* and *M. reinhardti* (Characiformes: Anostomidae). Genetica. 2024. 10.1007/s10709-024-00206-3. 38587599 10.1007/s10709-024-00206-3

[36] Ota K, Tateno Y, Gojobori T. Highly differentiated and conserved sex chromosome in fish species (*Aulopus japonicus*: teleostei, Aulopidae). Gene. 2003. 10.1016/S0378-1119(03)00702-9. 14604807 10.1016/s0378-1119(03)00702-9

[37] Yano CF, Sember A, Kretschmer R, Bertollo LAC, Ezaz T, Hatanaka T, et al. Against the mainstream: exceptional evolutionary stability of ZW sex chromosomes across the fish families Triportheidae and Gasteropelecidae (Teleostei: Characiformes). Chromosome Res. 2021. 10.1007/s10577-021-09674-1. 34694531 10.1007/s10577-021-09674-1

[38] Nirchio M, Oliveira C, Cioffi MB, Rossi FMC, Valdiviezo J, Paim FG et al. Occurrence of sex chromosomes in fish of the genus *Ancistrus* with a new description of multiple sex chromosomes in the Ecuadorian endemic *Ancistrus clementinae* (Loricariidae). Genes. 2023; 10.3390/genes14020306 10.3390/genes14020306 PMC9956960 36833233

[39] Nanda I, Volff JN, Weis S, Körting C, Froschauer A, Schmid M, et al. Amplification of a long terminal repeat-like element on the Y chromosome of the platyfish, *Xiphophorus maculatus*. Chromosoma. 2000. 10.1007/s004120050425. 10929195 10.1007/s004120050425

[40] Terencio ML, Schneider CH, Gross MC, Nogaroto V, Almeida MC, Artoni RF, et al. Repetitive sequences associated with differentiation of W chromosome in *Semaprochilodus taeniurus*. Genetica. 2012. 10.1007/s10709-013-9699-4. 23325335 10.1007/s10709-013-9699-4

[41] Chalopin D, Volff JN, Galiana D, Anderson JL, Schartl M. Transposable elements and early evolution of sex chromosomes in fish. Chromosome Res. 2015. 10.1007/s10577-015-9490-8. 26429387 10.1007/s10577-015-9490-8

[42] Utsunomia R, Silva DMZA, Ruiz-Ruano FJ, Goes CAG, Melo S, Ramos LP, et al. Satellitome landscape analysis of *Megaleporinus macrocephalus* (Teleostei, Anostomidae) reveals intense accumulation of satellite sequences on the heteromorphic sex chromosome. Sci Rep. 2019. 10.1038/s41598-019-42383-8. 30971780 10.1038/s41598-019-42383-8 PMC6458115

[43] Kretschmer R, Goes CAG, Bertollo LAC, Ezaz T, Porto-Foresti F, Toma GA, et al. Satellitome analysis illuminates the evolution of ZW sex chromosomes of Triportheidae fishes (Teleostei: Characiformes). Chromosoma. 2022. 10.1007/s00412-022-00768-1. 35099570 10.1007/s00412-022-00768-1

[44] Štundlová J, Hospodářská M, Lukšíková K, Voleníková A, Pavlica T, Altmanová M, et al. Sex chromosome differentiation via changes in the Y chromosome repeat landscape in African annual killifishes *Nothobranchius furzeri* and *N. kadleci*. Chromosome Res. 2022. 10.1007/s10577-022-09707-3. 36208359 10.1007/s10577-022-09707-3

[45] de Moraes RLR, Sassi FMC, Vidal JAD, Goes CAG, Santos RZ, Stornioli JHF, et al. Chromosomal rearrangements and satellite DNAs: extensive chromosome reshuffling and the evolution of neo-sex chromosomes in the genus *Pyrrhulina* (Teleostei; Characiformes). Int J Mol Sci. 2023. 10.3390/ijms241713654. 37686460 10.3390/ijms241713654 PMC10563077

[46] Lukšíková K, Pavlica T, Altmanová M, Štundlová J, Pelikánová Š, Simanovsky SA, et al. Conserved satellite DNA motif and lack of interstitial telomeric sites in highly rearranged African *Nothobranchius* killifish karyotypes. J Fish Biol. 2023. 10.1111/jfb.15550. 37661806 10.1111/jfb.15550

[47] Deon GA, dos Santos RZ, Sassi FDMC, Moreira-Filho O, Vicari MR, Porto-Foresti F, et al. The role of satellite DNAs in the chromosomal rearrangements and the evolution of the rare XY_1_Y_2_ sex system in *Harttia* (Siluriformes: Loricariidae). J Hered. 2024. 10.1093/jhered/esae028. 38757192 10.1093/jhered/esae028

[48] Toma GA, Sember A, Goes CAG, Kretschmer R, Porto-Foresti F, Bertollo LAC, et al. Satellite DNAs and the evolution of the multiple X_1_X_2_Y sex chromosomes in the wolf fish *Hoplias malabaricus* (Teleostei; Characiformes). Sci Rep. 2024. 10.1038/s41598-024-70920-7. 39223262 10.1038/s41598-024-70920-7 PMC11369246

[49] Herpin A, Braasch I, Kraeussling M, Schmidt C, Thoma EC, Nakamura S, et al. Transcriptional rewiring of the sex determining *dmrt1* gene duplicate by transposable elements. PLoS Genet. 2010. 10.1371/journal.pgen.1000844. 20169179 10.1371/journal.pgen.1000844 PMC2820524

[50] Schartl M, Schories S, Wakamatsu Y, Nagao Y, Hashimoto H, Bertin C, et al. *Sox5* is involved in germ-cell regulation and sex determination in medaka following co-option of nested transposable elements. BMC Biol. 2018. 10.1186/s12915-018-0485-8. 29378592 10.1186/s12915-018-0485-8 PMC5789577

[51] Wang L, Sun F, Wan Z, Yang Z, Tay YX, Lee M, et al. Transposon-induced epigenetic silencing in the X chromosome as a novel form of *dmrt1* expression regulation during sex determination in the fighting fish. BMC Biol. 2022. 10.1186/s12915-021-01205-y. 34996452 10.1186/s12915-021-01205-y PMC8742447

[52] Bertho S, Herpin A, Schartl M, Guiguen Y. Lessons from an unusual vertebrate sex-determining gene. Phil Trans R Soc B: Biol Sci. 2021. 10.1098/rstb.2020.0092. 10.1098/rstb.2020.0092 PMC8273500 34247499

[53] Plohl M, Meštrović N, Mravinac B. Satellite DNA Evolution. *In Genome Dynamics*; 2012. pp. 126–152. 10.1159/000337122 22759817

[54] Garrido-Ramos MA, Satellite DNA: an evolving topic. Genes. 2017. 10.3390/genes8090230. 10.3390/genes8090230 PMC5615363 28926993

[55] Šatović-Vukšić E, Plohl M. Satellite DNAs– from localized to highly dispersed genome components. Genes. 2023. 10.3390/genes14030742. 36981013 10.3390/genes14030742 PMC10048060

[56] Crepaldi C, Parise-Maltempi PP. Heteromorphic sex chromosomes and their DNA content in fish: an insight through satellite DNA accumulation in *Megaleporinus elongatus*. Cytogenet Genome Res. 2020. 10.1159/000506265. 32092756 10.1159/000506265

[57] de Oliveira MPB, Kretschmer R, Deon GA, Toma GA, Ezaz T, Goes CAG, et al. Following the pathway of W chromosome differentiation in *Triportheus* (Teleostei: Characiformes). Biology. 2023. 10.3390/biology12081114. 37626998 10.3390/biology12081114 PMC10452202

[58] Crepaldi C, Martí E, Gonçalves ÉM, Martí DA, Parise-Maltempi PP. Genomic differences between the sexes in a fish species seen through satellite DNAs. Front Genet. 2021. 10.3389/fgene.2021.728670. 34659353 10.3389/fgene.2021.728670 PMC8514694

[59] Toma GA, dos Santos N, dos Santos R, Rab P, Kretschmer R, Ezaz T, et al. Cytogenetics meets genomics: cytotaxonomy and genomic relationships among color variants of the Asian arowana *Scleropages formosus*. Int J Mol Sci. 2023. 10.3390/ijms24109005. 37240350 10.3390/ijms24109005 PMC10219274

[60] Ruiz-Ruano F, López-León M, Cabrero J, Camacho JPM. High-throughput analysis of the satellitome illuminates satellite DNA evolution. Sci Rep. 2016. 10.1038/srep28333. 27385065 10.1038/srep28333 PMC4935994

[61] Bardella VB, Milani D, Cabral-de-Mello DC. Analysis of *Holhymenia histrio* genome provides insight into the SatDNA evolution in an insect with holocentric chromosomes. Chromosome Res. 2020. 10.1007/s10577-020-09642-1. 32951078 10.1007/s10577-020-09642-1

[62] Cabral-de-Mello DC, Marec F. Universal fluorescence *in situ* hybridization (FISH) protocol for mapping repetitive DNAs in insects and other arthropods. Mol Genet Genomics. 2021. 10.1007/s00438-021-01765-2. 33625598 10.1007/s00438-021-01765-2

[63] Stornioli JHF, Goes CAG, Calegari RM, dos Santos RZ, Giglio LM, Foresti F, et al. The B chromosomes of *Prochilodus lineatus* (Teleostei, Characiformes) are highly enriched in satellite DNAs. Cells. 2021. 10.3390/cells10061527. 34204462 10.3390/cells10061527 PMC8235050

[64] Voleníková A, Lukšíková K, Mora P, Pavlica T, Altmanová M, Štundlová J, et al. Fast satellite DNA evolution in *Nothobranchius* annual killifishes. Chromosome Res. 2023. 10.1007/s10577-023-09742-8. 37985497 10.1007/s10577-023-09742-8 PMC10661780

[65] Silva DMZA, Utsunomia R, Ruiz-Ruano FJ, Daniel SN, Porto-Foresti F, Hashimoto DT, et al. High-throughput analysis unveils a highly shared satellite DNA library among three species of fish genus *Astyanax*. Sci Rep. 2017. 10.1038/s41598-017-12939-7. 29018237 10.1038/s41598-017-12939-7 PMC5635008

[66] Serrano-Freitas ÉA, Silva DMZA, Ruiz-Ruano FJ, Utsunomia R, Araya-Jaime C, Oliveira C, et al. Satellite DNA content of B chromosomes in the characid fish *Characidium gomesi* supports their origin from sex chromosomes. Mol Genet Genomics. 2020. 10.1007/s00438-019-01615-2. 31624915 10.1007/s00438-019-01615-2

[67] Biscotti MA, Olmo E, Heslop-Harrison JS. Repetitive DNA in eukaryotic genomes. Chromosome Res. 2015. 10.1007/s10577-015-9499-z. 26514350 10.1007/s10577-015-9499-z

[68] Camacho JPM, Cabrero J, Lopez-Leon MD, Martín-Peciña M, Perfectti F, Garrido-Ramos MA, et al. Satellitome comparison of two oedipodine grasshoppers highlights the contingent nature of satellite DNA evolution. BMC Biol. 2022. 10.1186/s12915-021-01216-9. 35130900 10.1186/s12915-021-01216-9 PMC8822648

[69] Schartl M, Schmid M, Nanda I. Dynamics of vertebrate sex chromosome evolution: from equal size to giants and dwarfs. Chromosoma. 2016. 10.1007/s00412-015-0569-y. 26715206 10.1007/s00412-015-0569-y

[70] Müller S, Du K, Guiguen Y, Pichler M, Nakagawa S, Stöck M, et al. Massive expansion of sex-specific SNPs, transposon-related elements, and neocentromere formation shape the young W-chromosome from the mosquitofish *Gambusia affinis*. BMC Biol. 2023. 10.1186/s12915-023-01607-0. 10.1186/s12915-023-01607-0 PMC10186657 37189152

[71] Voleníková AC, Sahara K, Štundlová J, Dalíková M, Koutecký P, Grof-Tisza P, et al. Ghost W chromosomes and unique genome architecture in ghost moths of the family Hepialidae. bioRxiv. 2023. 10.1101/2023.09.03.556148.38106088

[72] Soh YS, Alföldi J, Pyntikova T, Brown LG, Graves T, Minx PJ, et al. Sequencing the mouse Y chromosome reveals convergent gene acquisition and amplification on both sex chromosomes. Cell. 2014. 10.1016/j.cell.2014.09.052. 25417157 10.1016/j.cell.2014.09.052 PMC4260969

[73] Bachtrog D, Mahajan S, Bracewell R. Massive gene amplification on a recently formed *Drosophila* Y-chromosome. Nat Ecol Evol. 2019. 10.1038/s41559-019-1009-9. 31666742 10.1038/s41559-019-1009-9 PMC7217032

[74] Vegesna R, Tomaszkiewicz M, Ryder OA, Campos-Sánchez R, Medvedev P, DeGiorgio M, et al. Ampliconic genes on the great ape Y chromosomes: rapid evolution of copy number but conservation of expression levels. Genome Biol Evol. 2020. 10.1093/gbe/evaa088. 32374870 10.1093/gbe/evaa088 PMC7313670

[75] Zhou R, Macaya-Sanz D, Carlson CH, Schmutz J, Jenkins JW, Kudrna D, et al. A willow sex chromosome reveals convergent evolution of complex palindromic repeats. Genome Biol. 2020. 10.1186/s13059-020-1952-4. 32059685 10.1186/s13059-020-1952-4 PMC7023750

[76] Conte MA, Clark FE, Roberts RB, Xu L, Tao W, Zhou Q, et al. Origin of a giant sex chromosome. Mol Biol Evol. 2021. 10.1093/molbev/msaa319. 33300980 10.1093/molbev/msaa319 PMC8042771

[77] Rejón CR, Jamilena M, Garrido Ramos M, Parker JS, Ruiz Rejón M. Cytogenetic and molecular analysis of the multiple sex chromosome system of *Rumex acetosa*. J Hered. 1994. 10.1038/hdy.1994.28.

[78] Mestriner CA, Galetti PM, Valentini SR, Ruiz IRG, Abel LDS, Moreira-Filho O, et al. Structural and functional evidence that a B chromosome in the characid fish *Astyanax scabripinnis* is an isochromosome. J Hered. 2000. 10.1046/j.1365-2540.2000.00702.x. 10.1046/j.1365-2540.2000.00702.x 10971685

[79] Artoni RF, Vicari MR, Endler AL, Cavallaro ZI, de Jesus CM, Almeida MC, et al. Banding pattern of A and B chromosomes of *Prochilodus lineatus* (Characiformes, Prochilodontidae), with comments on B chromosomes evolution. Genetica. 2006. 10.1007/s10709-005-4846-1. 16850231 10.1007/s10709-005-4846-1

[80] Poletto AB, Ferreira IA, Martins C. The B chromosomes of the African cichlid fish *Haplochromis obliquidens* harbour 18S rRNA gene copies. BMC Genet. 2010. 10.1186/1471-2156-11-1. 20051104 10.1186/1471-2156-11-1 PMC2806386

[81] dos Santos LP, Francisco CM, Campos Júnior EO, Castro JP, Utsunomia R, Morelli S, et al. Chromosomal instability and origin of B chromosomes in the Amazonian glass tetra *Moenkhausia oligolepis* (Günther, 1864) (Characiformes, Characidae). Cytogenet Genome Res. 2021. 10.1159/000517091. 34433167 10.1159/000517091

[82] Kasiroek W, Indananda C, Luangoon N, Pinthong K, Supiwong W, Tanomtong A. First chromosome analysis of the humpback cardinalfish, *Fibramia lateralis* (Perciformes, Apogonidae). Cytologia. 2017. 10.1508/cytologia.82.9.

[83] Supiwong W, Tanomtong A, Pinthong K, Kaewmad P, Poungnak P, Jangsuwan N. The first chromosomal characteristics of nucleolar organizer regions and karyological analysis of Pink anemonefish, *Amphiprion perideraion* (Perciformes, Amphiprioninae). Cytologia. 2015. 10.1508/cytologia.80.271.

[84] Sumner AT. A simple technique for demonstrating centromeric heterochromatin. Exp Cell Res. 1972. 10.1016/0014-4827(72)90558-7. 4117921 10.1016/0014-4827(72)90558-7

[85] Sambrook J, Russel DW. Molecular cloning: a laboratory manual. Cold Spring Harbor; 2001.

[86] Novák P, Robledillo LA, Koblížková A, Vrbová I, Neumann P, Macas J. TAREAN: a computational tool for identification and characterization of satellite DNA from unassembled short reads. Nucleic Acids Res. 2017. 10.1093/nar/gkx257. 28402514 10.1093/nar/gkx257 PMC5499541

[87] Bolger AM, Lohse M, Usadel B, Trimmomatic. A flexible trimmer for illumina sequence data. Bioinformatics. 2014. 10.1093/bioinformatics/btu170. 10.1093/bioinformatics/btu170 PMC4103590 24695404

[88] Schmieder R, Edwards R. Fast identification and removal of sequence contamination from genomic and metagenomic datasets. PLoS ONE. 2011. 10.1371/journal.pone.0017288. 21408061 10.1371/journal.pone.0017288 PMC3052304

[89] Smit AFA, Hubley R, Green P. RepeatMasker Open-3.0 1996–2010. 2017.

[90] Ijdo JW, Wells RA, Baldini A, Reeders ST. Improved telomere detection using a telomere repeat probe (TTAGGG)_n_ generated by PCR. Nucleic Acids Res. 1991. 10.1093/nar/19.17.4780. 1891373 10.1093/nar/19.17.4780 PMC328734

[91] Sassi FMC, Toma GA, Cioffi MB. FISH-in fish chromosomes. Cytogenetics and molecular cytogenetics. CRC: Boca Raton 2022. pp. 281–97.

[92] Zwick MS, Hanson RE, Islam-Faridi MN, Stelly DM, Wing RA, Price HJ, et al. A rapid procedure for the isolation of *C*_0_*t*-1 DNA from plants. Genome. 1997. 10.1139/g97-020. 18464813 10.1139/g97-020

[93] Sember A, Bertollo LAC, Ráb P, Yano CF, Hatanaka T, Oliveira EA, et al. Sex chromosome evolution and genomic divergence in the fish *Hoplias malabaricus* (Characiformes, Erythrinidae). Front Genet. 2018. 10.3389/fgene.2018.00071. 29556249 10.3389/fgene.2018.00071 PMC5845122

